# Clinical Assessment of Stereoacuity and 3-D Stereoscopic Entertainment

**DOI:** 10.3109/09273972.2015.1107600

**Published:** 2015-12-15

**Authors:** Laurence P. Tidbury, Robert H. Black, Anna R. O’Connor

**Affiliations:** ^a^ Directorate of Orthoptics and Vision Science, and Department of Psychological Sciences, University of Liverpool, Liverpool, United Kingdom; ^b^ Department of Psychological Sciences, University of Liverpool, Liverpool, United Kingdom; ^c^ Directorate of Orthoptics and Vision Science, University of Liverpool, Liverpool, United Kingdom

**Keywords:** Binocular vision, depth perception, diagnostic tests, stereopsis, vision, visual perception

## Abstract

*Background/Aims*: The perception of compelling depth is often reported in individuals where no clinically measurable stereoacuity is apparent. We aim to investigate the potential cause of this finding by varying the amount of stereopsis available to the subject, and assessing their perception of depth viewing 3-D video clips and a Nintendo 3DS.

*Methods*: Monocular blur was used to vary interocular VA difference, consequently creating *4* levels of measurable binocular deficit from normal stereoacuity to suppression. Stereoacuity was assessed at each level using the TNO, Preschool Randot®, Frisby, the FD2*,* and Distance Randot®. Subjects also completed an object depth identification task using the Nintendo 3DS, a static 3DTV stereoacuity test*,* and a 3-D perception rating task of *6* video clips.

*Results*: As intraocular VA differences increased, stereoacuity of the 57 subjects (aged 16–62 years) decreased (eg*,* 110”, 280”, 340”*,* and suppression). The ability to correctly identify depth on the Nintendo 3DS remained at 100% until suppression of one eye occurred. The perception of a compelling 3-D effect when viewing the video clips was rated high until suppression of one eye occurred, where the 3-D effect was still reported as fairly evident.

*Conclusion*: If an individual has any level of measurable stereoacuity, the perception of 3-D when viewing stereoscopic entertainment is present. The presence of motion in stereoscopic video appears to provide cues to depth, where static cues are not sufficient. This suggests there is a need for a dynamic test of stereoacuity to be developed, to allow fully informed patient management decisions to be made.

## Introduction

A large proportion of the population has binocular vision deficits, commonly caused by strabismus, with a prevalence between 2.3% and 3.6% in young children alone^6^^,^ 12 ^,^ 14 ^,^ 24 The lack of appreciation of stereopsis has the potential to impact in a number of ways, eg, children not being able to participate in three-dimensional (3-D) films and games, a potential detriment to education^4^ and employment prospects. In addition, stereopsis is beneficial for the completion of “real world” tasks,^3^^,^ 9 ^,^ 16 ^,^ 20 supporting the need for treatment to maximize binocular vision.

In recent years there has been a large increase in access to 3-D film, television, and gaming technology. This has been accompanied by anecdotal reports^1^^,^ 2 and small-scale studies^22^ of individuals diagnosed as stereoblind by clinical measures, describing the ability to perceive stereoscopic depth during or following exposure to 3-D entertainment media. This apparent contradiction between clinical findings and reported perception could originate solely from monocular cues^22^ which are present in entertainment media, and not controlled as in clinical testing. The reliance on anecdotal reports and studies with small sample sizes and large intersubject variability does not allow a clear conclusion to be made on how a person might perceive 3-D with diminished or absent stereoacuity.

The aim of the present study was to evaluate the relationship between clinical measures of stereoacuity and the perception of depth in 3-D entertainment media. By comparing the performance of the same subject under artificially varied stereoacuity levels,^17^^,^ 18 a reliable comparison between varying levels of stereoacuity can be established, standardizing the utilisation of monocular cues to depth in the scene.

## Materials and Methods

### Subjects

Ethical approval was gained from the University of Liverpool Ethics Sub-committee and the study was performed in accordance with the ethical standards laid down in the Declaration of Helsinki. Informed consent was gained prior to participation. The inclusion criteria were for subjects aged above 16 years, with vision in one eye of at least 0.22 LogMAR with a difference of less than 0.2 logMAR between the eyes, and with measurable stereoacuity, in order to exclude those with amblyopia and stereoblindness. Visual acuity (VA) of each eye was measured using an ETDRS (Precision Vision) chart at 3 m. No similar research was found to facilitate sample size calculation, therefore the study aimed to recruit a convenience sample of 50 subjects. All testing was performed with habitual correction.

### Creation of Varying Stereoacuity Levels

The subject’s dominant eye (determined by subject choice using the pointing method) was temporarily occluded. Increasing strengths of convex lenses were added to the subject’s habitual correction of the nondominant eye by the researcher to establish the amount of blur necessary to create a difference in VA of 3 lines, then 6 lines, and until suppression of the blurred eye was achieved (tested with occlusion removed). This created 4 blurred states (A—no blur, B—slight blur, C—moderate blur, and D-suppression) of VA difference between the eyes, to artificially reduce stereoacuity. Sensory fusion was confirmed in the first 3 states and proved absent in state D using Bagolini glasses. We aimed for suppression rather than monocular testing, as this is more representative of amblyopic individuals.

### Tasks

Each of the following tasks were performed consecutively a total of 4 times, once in each blur state. The order of states and order of testing were randomized by computer for each task and subject.

### Clinical Measures of Stereoacuity

Near stereoacuity was assessed using TNO (Richmond Products), Randot® Preschool Stereotest (Stereo-Optical), and Frisby (Frisby Stereotest™). Distance stereoacuity was assessed using the FD2 (Frisby Stereotest™) and Distance Randot® (Stereo-Optical).

### Near Stereoscopic Task

Six scenes with features at varying levels of disparity were chosen from a Nintendo 3DS (Nintendo™) game. The 3DS was not presented at a fixed location; rather, the subject was free to move the device to mirror typical usage. Movement of the device has two main effects on the binocular depth cues; lateral movement can result in the image meant for the right eye being presented to the left eye, and variations in viewing distance result in an increase or decrease in the angle of disparity. Variations in these two factors may provide a dynamic cue to depth.

For each blur state, the subject was presented 3 out of the 6 scenes (in a randomized and balanced order). The subject had to determine the order in depth of selected features in the scene, ranked from 1 to 5 (closest to furthest). The subject was encouraged to use all cues in the scene to determine order of depth and was allowed to position the device as desired, replicating normal device use. If unable to determine a difference between objects, the subject was instructed to guess, or label the highlighted features with the same number. The percentage correct score was calculated for each picture by determining the number of objects identified in the correct order, out of the total number of objects.

### 3DTV Video Task

The 3DTV used was a 32”, 1360x768 pixel, passive, circular polarized screen. The TV was mounted on a height-adjustable table, 1.2 m from the subject, positioned with the center of the screen aligned horizontally and vertically with the subject’s eyes.

Subjects were primed to this task using two example video clips (habitual correction), with responses kept by the subject for later reference. The 3-D video task was graded using a Likert/Punum ladder scale.^5^ For every video clip, the same 10-point rating scale banded into 3 sections was used. Banding 1 to 3 contained the descriptor “3-D effect not really seen. Appears mostly 2-D”; 4 to 7 “3-D effect fairly evident”; and 8 to 10 “3-D effect very obvious, you feel you would need to move out of the way or catch objects from the screen.” For each blur state, 5 different 3-D video clips were presented in a randomized order.

A subset of subjects tested later into the study, had a random selection (2 of 5 videos in each state) of the video clips played to them in monoscopic mode (2-D), achieved by displaying the right eye image to both eyes simultaneously. The subject was not informed as to the whether the content was presented in 3-D or 2-D.

### 3DTV Static Task

**Figure 1.**
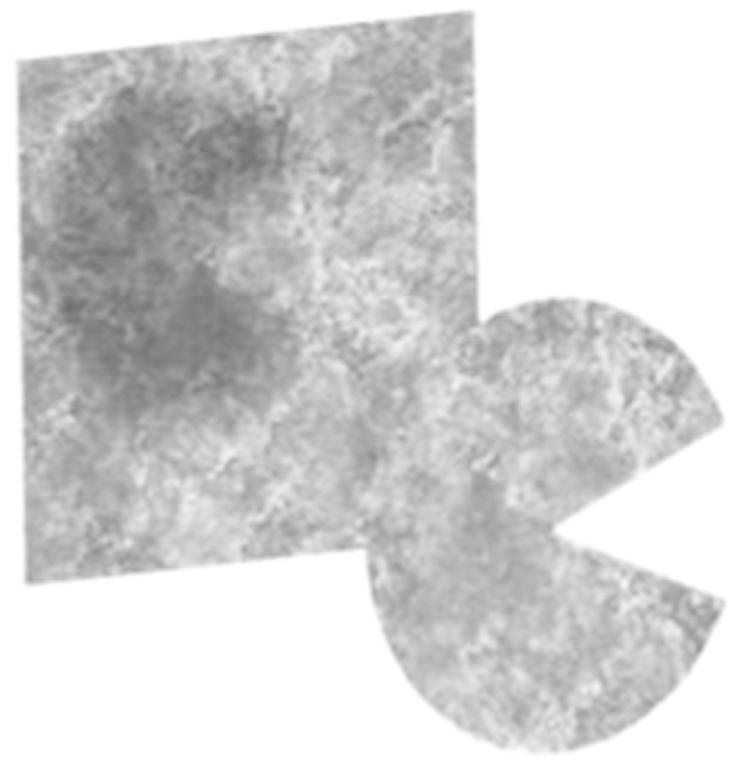
Monocularly viewable depiction of Pacman stimuli with crossed disparity (inclusion of shadow is indicative of depth and not present in actual stimuli).

A digital stereoacuity test was developed using a “Pacman” shaped stimulus of a random texture, presented in front of the same random textured background (Figure 1). By shifting the stimuli by a certain number of pixels, crossed disparity was created. Stereoacuity levels (1.64, 2.64, 3.21, 3.35, and 3.56 logArcSec) were chosen to cover a similar range to those present in the 3-D video clips.

In each blur state 7 trials were presented; 5 of these contained a stereoscopic pair, 1 negative control (no stimuli), and 1 positive control (a complete circle if simultaneous perception, or a single Pacman if suppression). For each presentation subjects were asked to identify the direction of the mouth.

## Statistical Analysis

Normality was determined using the Kolmogorov-Smirnov test. Mean ± standard deviation (SD) is presented if data were normally distributed and median ± interquartile range if not. As clinical tests of stereopsis measure distinct values of stereoacuity, with large intervals, the clinical tests and 3DTV static task were analyzed using non-parametric methods. For the purpose of analysis a value of 5 log arc seconds was assigned where no binocular vision was demonstrable and 4 log arc seconds if simultaneous perception was present.

Friedman’s test was used to assess the variability between stereoacuity or performance in each blur state, for each task. If a significant result was returned, post hoc analysis was performed using Wilcoxon’s signed ranks test. In the subset of subjects who were shown a mix of 2-D and 3-D video clips, the Mann-Whitney U test was performed as an uneven amount of 2-D and 3-D videos were shown. A total of 6 individual comparisons were possible in each task (4 blur states), therefore Bonferroni corrections were applied to maintain an α of 0.05. The corrected α value was therefore set at 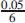, α = 0.0083.

## Results

Fifty-seven subjects were recruited from within the University of Liverpool aged 16–62 years, with an average (median [IQR]) VA of −0.10(0.16) in both eyes. The subjects had no manifest deviation and all had demonstrable stereoacuity on all 3 clinical near tests of stereopsis.

### States of Blur and Clinical Measures of Near and Distance Stereopsis Task

The right eye was blurred in 48% of subjects and the left blurred in 52%. Statistically significant (*P* < 0.001) overall differences were found between each state for each test and confirmed by post hoc analysis (*P* < 0.0083) (Table 1). This confirms that the monocular lens blur model effectively reduces stereoacuity.

**Table 1.** Results of the clinical stereoacuity tests, 3DTV Static Task (digital stereoacuity test), 3DTV video clip ratings, and the 3DS near task. The interocular visual acuity difference and lens power required to create each blur state is provided. The differences in stereoacuity scores between each blur state were statistically significantly different for the clinical stereoacuity tests and the 3DTV Static Task (*P* < 0.001) (*n* = 57).

	A (No Blur)	B (3 lines of blur)	C (6 lines of blur)	D (Suppression)
VA difference between eyes (*LogMAR) Median (IQR)*	0.0 (0.02)	0.30 (0.04)	0.67 (0.06)	1.09 (0.06)
Lens Required *(Dioptres) Median (IQR)*	Nil	+1.25 (0.50)	+2.00 (0.75)	+9.00 (3.0)
TNO *(LogArcSeconds) Median (IQR) Range*	1.78 (0.3) 1.18–5.00	2.08 (0.90) 1.48–5.00	3.29 (2.62) 1.48–5.00	5.00 (0) 5.00–5.00
Pre-School Randot *(LogArcSeconds) Median (IQR) Range*	1.60 (0.4) 1.30–5.00	2.00 (0.60) 1.60–5.00	5.00 (2.62) 1.78–5.00	5.00 (0) 5.00–5.00
Frisby *(LogArcSeconds) Median (IQR) Range*	1.30 (0.18) 1.30–5.00	1.88 (0.57) 1.30–5.00	2.20 (0.73) 1.30–5.00	5.00 (0) 1.93–5.00
Distance Randot *(LogArcSeconds) Median (IQR) Range*	1.78 (0.22) 1.30–5.00	5.00 (2.39) 1.78–5.00	5.00 (0.00) 2.60–5.00	5.00 (0) 5.00–5.00
FD2 *(LogArcSeconds) Median (IQR) Range*	1.18 (0.48) 0.7–5.00	5.00 (3.39) 1.3–5.00	5.00 (0.00) 1.70–5.00	5.00 (0) 5.00–5.00
3DTV Static Task *(LogArcSeconds) Median (IQR) Range*	1.94 (1.00) 1.94–4.00	2.94 (1.57) 1.94–4.00	3.80 (0.49) 1.94–5.00	5.00 (0.00) 5.00–5.00
3DTV Video Clips *(Rating out of 10) Median (IQR) Range*	9 (3) 1–10	8 (3) 1–10	7 (4) 1–10	4 (5) 1–10
Near 3DS Task *(Percentage Correct) Median (IQR)Range*	100 (25) 0–100	100 (25) 0–100	90 (60) 0–100	20 (60) 0–100

### 3DTV Static Task

All scores are shown in Table 1. There are statistically significant overall differences between all 4 states (*P* < 0.001) and post hoc individual comparisons were significant in all cases (*P* < 0.001), other than between state A and B.

### 3DTV Video Task

Statistically significant overall differences between all states were found for the 3DTV task (*P* < 0.001). Post hoc analysis shows that other than the individual comparison of states B and C (where the median rank difference is 0.5, CI 0–1.5, *P* = 0.036), all states were statistically significantly different to each other (*P* < 0.0083). The maximum and minimum amounts of crossed and uncrossed disparity were measured for each video (Table 2).

**Table 2.** Maximum crossed and uncrossed disparities of each video clip. The ratings provided by each subject are also provided to illustrate that it is not necessarily the range of disparities that provide the “3-D effect” (*n *= 57). The title of each column briefly describes the contents of the video clip.

	Baseball	Fish	Pole Vault	Fencing	Ribbon	Baseball
Max Crossed Disparity *(LogArcSeconds)*	3.1	2.88	3.31	3.56	2.81	3.1
Max Uncrossed Disparity *(LogArcSeconds)*	2.48	2.93	2.71	3.33	2.83	2.48
3DTV Video Clips – State A *(Rating out of 10) Median (IQR)*	9 (2)	7 (3)	9 (2)	9 (2)	8 (3)	9 (2)

### 2-D Video vs. 3-D Video Subset

A subset of 24 subjects was tested under monoscopic versus stereoscopic conditions (Table 3). For blur states A, B, and C the 3-D video clips were rated statistically significantly better than the 2-D clips (*P* < 0.001). In state D no significant difference was found (*P* = 0.711). While overall differences were present between blur states for the 2-D videos (*P* = 0.0033), post hoc analysis shows that this is driven by the only statistically significant difference, between states A and D (*P* > 0.0038). Ratings of the 3-D videos decreased as the amount of monocular blur increased (*P* < 0.001).

**Table 3.** Subject ratings for 3-D and 2-D video clips. Ratings were significantly better for the 3-D clips (*P* < 0.001), other than in state D, where no difference was found (*n* = 24).

	A (No blur)	B (3 lines of blur)	C (6 lines of blur)	D (Suppression)
3-D Video Clips *(Rating out of 10) Median (IQR) Range*	9 (3) 1–10	8 (3) 1–10	7 (5) 1–10	3 (5) 1–10
2-D Video Clips *(Rating out of 10) Median (IQR) Range*	4 (5.5) 1–10	3 (5) 1–10	4 (5) 1–10	3.5 (5) 1–10

### Near Stereoscopic Task (3DS)

The median (IQR) scores for this task shown in Table 1 have statistically significant overall differences (*P* < 0.001). Post hoc individual comparisons showed differences between all states (*P* < 0.001), except between states A and B (*P* = 0.71) where no difference in performance is present.

## Discussion

These findings support the anecdotal evidence of individuals reporting the perception of depth in the absence of measurable stereoacuity. With a decrease in monocular blur (and subsequent increase in stereoacuity), perception of depth increased. In the presence of any measureable stereoacuity, the subjects reported the impression of convincing depth when viewing the 3-D videos.

A limitation of previous reports is variability between subjects, requiring a large sample size to control. The methodology used here minimized the effect of intersubject variability by comparing performance within subjects. We demonstrate significant variability within subjects (between blur levels), appreciating the same level of monocular cues in all conditions. Although the blurring lens method is not wholly representative of amblyopia, as an amblyopic eye perceives a distorted image,^11^^,^ 19 it is effective for reducing stereoacuity levels,^17^^,^ 18 allowing us to investigate and quantify the effect of degraded stereoacuity. As the blur model was determined at 3 m, there is a possibility that VA was different for near testing; this is not however a concern. Regardless of whether VA degradation was the same as stereoacuity degradation, we have demonstrated stereoacuity levels were reduced by our methodological choice of using this model.

Accurate performance of the 3DS task in the presence of reduced stereoacuity may be attributable to monocular cues; however, the drop to chance performance when one eye was suppressed suggests that binocular cues were being used to identify depth. Contrary to this, the perception of depth reduced with any reduction in stereoacuity when viewing 3-D video clips. However, some perception of depth was maintained when one eye was suppressed.

In this study the key variable that differs between clinical tests and entertainment media is the presence of dynamic elements. These dynamic elements take the form of either motion across the screen, changes in the amount of depth presented, and changes in shape or pattern. Other studies have reported similar findings, where patients with no measurable stereoacuity demonstrated the ability to perceive a 3-D effect if the stimulus was moving^7^^,^ 10 ^,^ 13 ^,^ 23 Motion-in-depth was integral within the video clips, however, the 3DS scenes displayed fixed disparities. Despite the lack of motion on the 3DS display, changing the viewing distance changes the amount of disparity presented, with lateral movements reversing the disparity direction entirely, resulting in a 2-frame displacement in depth of up to 3.31 logarcseconds (2063”). Irrespective of how motion-in-depth is generated, its presence appears to aid the detection of depth. It also acts to increase the amount of depth perceived in a scene versus fixed disparity; individuals feel a greater amount of depth is present when disparity increases, even if the total amount of disparity is the same as in a static presentation.^21^

Motion-in-depth is perceived though two separate mechanisms, detecting changing disparity over time and the recognition of intraocular velocity differences.^8^ It appears that these mechanisms are used to different extents by individuals,^15^ which could account for the large variability in the perception of depth with reduced static stereoacuity levels.

During the initial phase of this study, subjects reported higher levels of depth than anticipated when rating the 3-D videos. We hypothesized that this may have been attributable to monocular cues and to investigate this, some of the 3-D video clips were presented in 2-D only by displaying the right half-image to both eyes simultaneously. The subjects consistently provided a poorer rating for the 2-D videos, showing that binocular cues exceed the depth information provided by monocular cues to depth. When 3-D videos were viewed with monocular suppression, the subjects rated the 2-D video statistically significantly higher than the 3-D video. It would be beneficial to test a larger number of subjects to ensure the validity of these findings and to assess any impact of a reduction in binocular summation. The blur model, aside from reducing stereoacuity (as shown by the worsening thresholds using clinical assessment), reduced visual acuity, which in turn reduces the discernibility of the monocular cues to depth. We could have assessed this by reducing the resolution by the same degree in the stereoscopic condition (state A) and comparing with the suppression state (D).

A potential source of an improved perception of depth may have resulted from the method of displaying the stimuli. However, the results of the 3DTV test of static stereoacuity were very similar to the results of the clinical stereopsis assessment methods, under the varying amounts of blur. This suggests the display technology (backlighting, circular polarisation, lower resolution, etc) were not the reasons for perception of depth during the video presentation. Also, levels of stereoacuity assessed were greater than the range of disparities present in the video clips, suggesting that the amount of disparity is not why depth was perceived in the 3-D videos. Table 2 shows that the highest ratings were not always given to the videos with the most disparity.

Clinicians are frequently asked what the results of a stereoacuity test mean in relation to 3-D entertainment. These data suggest that the best advice is if there is any measurable stereoacuity, the perception of 3-D when viewing stereoscopic entertainment will be present. Even if no stereoacuity is measurable in the clinic, these results along with previous findings^22^ suggest that some degree of depth perception may be possible when viewing 3-D video. The findings of this study suggest that there is a need for a dynamic test of stereoacuity and a larger ranged static stereoacuity test to be developed, to allow full assessment of patients with binocular vision problems.

## References

[CIT0001] Arnold RW, Davidson S, Madigan WP (2011). Stereopsis and 3-D movies. J Pediatr Ophthalmol Strabismus.

[CIT0002] Barry SR (2009). Fixing My Gaze.

[CIT0003] Brown K, Buckley D (2004). Do we really need binocular single vision?. Br Ir Orthopt J.

[CIT0004] Brown PM, Hamilton NM, Denison AR (2012). A novel 3D stereoscopic anatomy tutorial. Clin Teach.

[CIT0005] Fletcher KE, French CT, Irwin RS, Corapi KM (2010). A prospective global measure, the punum ladder, provides more valid assessments of quality of life than a retrospective transition measure. J Clin Epidemiol.

[CIT0006] Friedman DS, Repka MX, Katz J (2009). Prevalence of amblyopia and strabismus in white and African American children aged 6 through 71 months the Baltimore Pediatric Eye Disease Study. Ophthalmology.

[CIT0007] Fujikado T, Hosohata J, Ohmi G (1998). Use of dynamic and colored stereogram to measure stereopsis in strabismic patients. Jpn J Ophthalmol.

[CIT0008] Harris JM, Nefs HT, Grafton CE (2008). Binocular vision and motion-in-depth. Spat Vis.

[CIT0009] Joy S, Davis H, Buckley D (2001). Is stereopsis linked to hand-eye coordination?. Br Orthopt J.

[CIT0010] Maeda M, Sato M, Ohmura T, Miyazaki Y (1999). Binocular depth-from-motion in infantile and late-onset esotropia patients with poor stereopsis. Invest Ophthalmol Vis Sci.

[CIT0011] Mansouri B, Hansen BC, Hess RF (2009). Disrupted retinotopic maps in amblyopia. Invest Ophthalmol Vis Sci.

[CIT0012] McKean-Cowdin R, Cotter SA, Tarczy-Hornoch K (2013). Prevalence of amblyopia or strabismus in Asian and non-Hispanic white preschool children: multi-ethnic pediatric eye disease study. Ophthalmology.

[CIT0013] Mollenhauer KA, Haase W (2000). Preliminary report: dynamic stereopsis in patients with impaired binocular function. Strabismus.

[CIT0014] Multi-ethnic Pediatric Eye Disease Study Group (2008). Prevalence of amblyopia and strabismus in African American and Hispanic children ages 6 to 72 months the multi-ethnic pediatric eye disease study. Ophthalmology.

[CIT0015] Nefs HT, O'Hare L, Harris JM (2010). Two independent mechanisms for motion-in-depth perception: Evidence from individual differences. Front Psychol.

[CIT0016] O'Connor AR, Birch EE, Anderson S, Draper H (2010). The functional significance of stereopsis. Invest Ophthalmol Vis Sci.

[CIT0017] O'Connor AR, Birch EE, Anderson S, Draper H (2010). Relationship between binocular vision, visual acuity, and fine motor skills. Optom Vis Sci.

[CIT0018] Piano ME, O'Connor AR (2013). The effect of degrading binocular single vision upon fine motor skill task performance. Invest Ophthalmol Vis Sci.

[CIT0019] Pugh M (1958). Visual distortion in amblyopia. Br J Ophthalmol.

[CIT0020] Read JCA, Begum SF, McDonald A, Trowbridge J (2013). The binocular advantage in visuomotor tasks involving tools. I-Perception.

[CIT0023] Watanabe Y, Kezuka T, Harasawa K, Usui M (2008). A new method for assessing motion-in-depth perception in strabismic patients. Br J Ophthalmol.

[CIT0024] Williams C, Northstone K, Howard M (2008). Prevalence and risk factors for common vision problems in children: data from the ALSPAC study. The Br J Ophthalmol.

